# Effects of Stress States and Joint Configurations on Dynamic Mechanical Properties of Rock Masses

**DOI:** 10.3390/ma18081699

**Published:** 2025-04-09

**Authors:** Tingting Liu, Zi Wang, Xuyi Wang, Shenghao Yang, Wenxu Huang, Luyang Ding

**Affiliations:** 1 School of Civil Engineering and Architecture, Wuhan University of Technology, Wuhan 430070, China; ttliu@whut.edu.cn (T.L.);; 2 Sanya Science and Education Innovation Park, Wuhan University of Technology, Sanya 572025, China

**Keywords:** Hopkinson pressure bar, continuous–discrete coupling method, flat-joint model, jointed rock

## Abstract

In complex geological environments, the discontinuous dynamic response behavior of jointed rock masses under the coupled effects of in situ stress and transient dynamic disturbances significantly exacerbates the risk of surrounding rock instability. This study establishes three-dimensional numerical models of various jointed rocks under uniaxial–biaxial–triaxial split Hopkinson pressure bar (SHPB) experimental systems through the coupling of the finite difference method (FDM) and discrete element method (DEM). The models adhere to the one-dimensional stress wave propagation assumption and satisfy the dynamic stress equilibrium requirements, demonstrating dynamic mechanical responses consistent with physical experiments. The results reveal that the synergistic–competitive effects between joint configuration and initial pre-compression jointly dominate the dynamic mechanical response of rocks. Multiaxial pre-compression promotes the development of secondary force chain networks, enhances rock impact resistance through multi-path stress transfer mechanisms, significantly improves strain energy storage density during peak stages, and drives failure modes to evolve from macroscopic through-going fractures to localized crushing zones. The spatial heterogeneity of joint configurations induces anisotropic characteristics in principal stress fabric. Single joint systems maintain structural integrity due to restricted weak plane propagation, while cross/parallel joints exhibit geometrically induced synergistic propagation effects, forming differentiated crack propagation paths that intensify frictional and kinetic energy dissipation. Through cross-scale numerical model comparisons, the evolution of force chain fabric, particle displacement distribution, microcrack propagation, and energy dissipation mechanisms were analyzed, unveiling the synergistic regulatory effects of the stress state and joint configuration on the rock dynamic response. This provides a theoretical basis for impact-resistant structure optimization and dynamic instability early warning in deep engineering projects involving jointed surrounding rock.

## 1. Introduction

**Figure 1:**
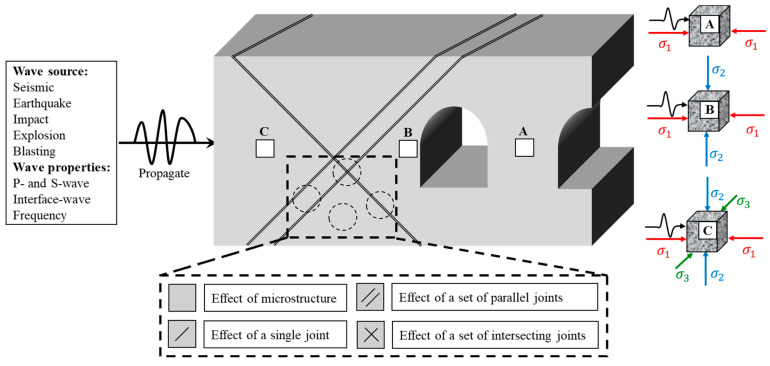
Dynamic response mechanisms of jointed rock masses in underground engineering (revised after Zhang and Zhao [5]).

Natural rock masses frequently contain joints and fissures due to geological tectonic activities and weathering, resulting in a reduction of integrity and structural discontinuities [1]. Due to the combined effects of in situ stress and external dynamic loads (such as mechanical vibrations, explosions, impacts, and earthquakes), the mechanical response of rock masses is controlled by the geometric morphology and spatial distribution of joints and fissures, adversely affecting the safety and stability of underground engineering structures [2]. As illustrated in Figure 1, in complex geological environments, joint systems commonly exhibit various spatial configurations, including single joints, cross joints, and parallel counterparts. The secondary stress field adjustment induced by underground engineering excavation unloading results in significant variations for the stress state of the surrounding rock [3,4]. Deep rock masses unaffected by excavation disturbances remain in a triaxial stress state ($\sigma_{1}\geq \sigma_{2}\geq \sigma_{3}$). The rock mass at the exposed face of the underground chamber is in a biaxial stress state ($\sigma_{1}\geq \sigma_{2}>\sigma_{3}=0$) due to excavation-induced unloading. Critical zones such as intermediate walls between adjacent chambers or mine pillars in stope areas experience a uniaxial stress state ($\sigma_{1}>\sigma_{2}=\sigma_{3}=0$). When superimposed with dynamic disturbances, the stress transmission path and failure mode are governed by the dominant joint configuration. Therefore, the synergistic mechanisms of multiple joint spatial configurations (single, cross, and parallel); diverse in situ stress fields (uniaxial, biaxial, and triaxial); and transient dynamic disturbances are of significant importance to reveal the dynamic processes of multi-mode failure in the rock mass. They also provide crucial theoretical support for the design of impact-resistant supports in deep underground engineering.

The evolution of stress paths and mechanical response characteristics of deep rock masses induced by excavation unloading are of importance in the prevention and control of deep engineering disasters. The classical rock mechanics theory originated from uniaxial compression tests, revealing the brittle failure characteristics, strength thresholds, and crack propagation laws of rocks [6,7]. As the underground engineering extends to deep space, conventional triaxial tests ($\sigma_{1}>\sigma_{2}=\sigma_{3}$) have become the primary schemes for investigating the multiaxial mechanical behavior of rock mass. By controlling the confining pressure ($\sigma_{2}=\sigma_{3}$), these tests systematically reveal the strengthening of rock strength with the increasing confining pressure, plastic deformation characteristics, and dilation properties, promoting the development of classical strength theories such as the Mohr–Coulomb criterion and Drucker–Prager model [8,9,10]. However, the in situ stress field in deep space frequently exhibits true triaxial non-equal pressure characteristics ($\sigma_{1}>\sigma_{2}>\sigma_{3}$) [11,12]. Breakthrough research based on true triaxial test platforms has revealed the regulatory mechanisms of intermediate principal stress on rock strength, failure modes, and energy release rates, correcting the prediction biases of traditional strength criteria for the mechanical behavior of deep rock [13]. Notably, the excavation unloading process leads to stress path reconstruction in the surrounding rock, where the rock near the free face transitions to a biaxial stress state ($\sigma_{1}>\sigma_{2},\;\sigma_{3}\to 0$) due to radial stress unloading [14,15]. This sudden change in stress state induces more tensile cracks, a faster post-peak stress drop, and more pronounced brittle characteristics. To address the static–dynamic coupling mechanical environment induced by excavation disturbances, researchers have innovatively integrated split Hopkinson pressure bar (SHPB) technology into multiaxial loading domains in recent years. By developing a triaxial SHPB collaborative loading system, dynamic impact tests on sandstone samples under multiaxial preload conditions have been achieved, successfully simulating the true stress state of deep rock masses subjected to the coupled action of static in situ stress and dynamic disturbance [16]. Multi-condition comparative investigations have indicated that dynamic strength, fragmentation energy consumption, and fracture modes exhibit constraint dependence. These innovations provide key experimental evidence for elucidating the mechanisms of dynamic disasters such as rock bursts and impact ground pressure in deep engineering, more accurately reproducing the mechanical response processes of deep engineering surrounding rock.

The development of joints and fissures weakens the load-bearing capacity of the surrounding rock, and diverse joint spatial combination modes significantly alter the mechanisms of crack initiation and propagation, inducing differentiated rupture modes and mechanical response mechanisms. For rock samples with non-through single joints, uniaxial compression tests based on DIC technology have revealed that prefabricated joints cause a degradation of rock material mechanical properties, with the degree of degradation reflected by the strain localization time history near the joint, as the joint inclination angle varies [17]. The mechanical laws of single-jointed rocks under dynamic impact are similar to those induced by static loading. The dynamic compressive strength gradually decreases as the joint inclination angle decreases or joint persistence increases [18], and the samples are more fragmented at high strain rates [19]. For the complex damage behavior of cross-jointed rocks, existing research through refined experiments has revealed that the geometric configuration of the primary and secondary joint planes (*α* angle and *β* angle) affects the uniaxial compressive strength (UCS) by regulating the tendency for shear failure [20]. In uniaxial dynamic impact tests on cross-jointed samples, through-going cross joints exhibit splitting failure modes [21], while non-through-going cross joints show “X”-shaped shear failure modes [22]. For rocks with parallel joints, the mutual constraint between joints easily induces a rock bridge through-going rupture. Static–dynamic combined loading tests on granite with parallel double fissures, combined with DIC full-field strain monitoring, have revealed deformation and failure characteristics and crack evolution laws: parallel fissures initiate tensile–shear composite failure through the principal strain or shear strain concentration at the fissure tips, with the fissure inclination angle dominating the expansion path of the tensile strain zone [23].

At the research methodology level, numerical simulation technology has opened up new paths for multi-scale analysis of rock dynamic mechanical behavior. Early studies constructed SHPB numerical models based on the ANSYS AUTODYN platform, quantifying the effects of lateral inertial confinement, end friction, and sample geometry (L/D ratio) on the dynamic increase factor (DIF) of granite, limestone, and tuff [24]. Discontinuous deformation analysis (DDA) has accurately characterized the evolution characteristics from crack initiation to the macroscopic rupture of rocks under dynamic loading [25]. Based on the particle flow code (PFC), the dynamic mechanical mechanisms of rock materials can be directly simulated at the mesoscopic scale, realistically reproducing the entire process of rock dynamic crushing failure through mesoscopic-scale particle displacement vectors, contact force chains, and microcrack angles [26]. For multiaxial combined impact loading conditions, a three-dimensional continuous–discrete coupling method based on FLAC3D-PFC3D has been developed to construct a full-scale triaxial SHPB system. By simulating stress wave propagation in steel bars with continuous media and characterizing rock dynamic fracture behavior with discrete elements, rock dynamic damage evolution is accurately represented, and the computational efficiency is optimized [27].

This study addresses the insufficiency in systematically comparing the dynamic damage mechanisms of rock masses under the coupled effects of multi-joint spatial configurations (single/cross/parallel joints) and multidirectional initial stress states. It aims to reveal the multi-scale dynamic response patterns of jointed rock masses in deep underground complex stress–structure coupling environments. By establishing uniaxial–biaxial–triaxial Hopkinson pressure bar numerical models based on a continuous–discrete coupling (FDM-DEM) method, we innovatively conducted macro–meso collaborative analyses considering multiple joint types, diverse initial confinements, and dynamic impact loads. The research quantitatively compares dynamic strength characteristics, deformation features, force chain evolution patterns, crack propagation mechanisms, and energy dissipation laws. It systematically clarifies the synergistic–competitive effects between joint configurations and initial confinement environments in controlling rock mass dynamic instability. The pioneering establishment of a quantitative correlation model of “joint configuration–confinement path–dynamic damage” breaks through the limitations of traditional rock dynamics research that adopts “simplified single joint assumptions with uniaxial loading hypotheses”. This provides multi-scale criteria for the early warning of dynamic disasters in deep engineering rock masses, spanning from meso-scale damage accumulation to macro-scale instability evolution. The findings advance the theoretical development of nonlinear dynamic response mechanisms for rock masses under extreme geological conditions.

## 2. Numerical Method

### 2.1. Continuous–Discrete Coupling Method

**Figure 2:**
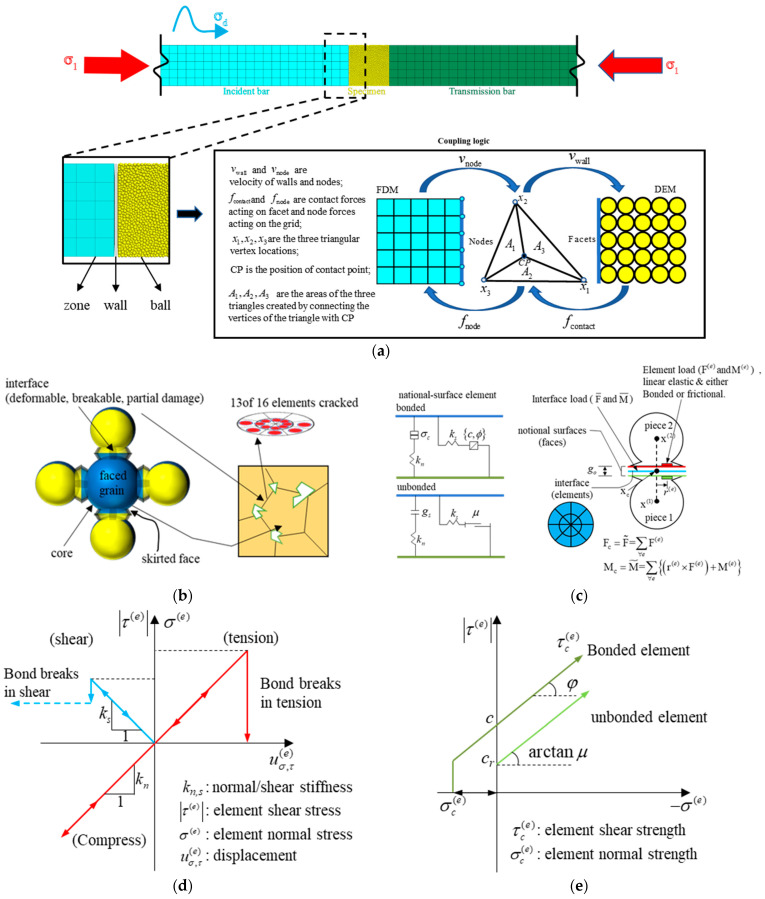
(**a**) Continuous–discrete coupled model and coupled logic under static and dynamic loads; (**b**) flat-jointed material; (**c**) behavior components of the flat-jointed model; (**d**) force–displacement law for a flat-jointed element; (**e**) failure envelope.

Based on the multi-scale coupling theory of continuous and discrete media, a full-size numerical model of the Hopkinson pressure bar system is constructed. As shown in Figure 2a, FEM is employed to establish three-dimensional continuous media models of the high-stiffness steel incident and transmitted bars, accurately characterizing the unattenuated propagation and reflection of elastic stress waves in the bar system; meanwhile, the particle DEM is used to construct the particle assembly of the rock sample by assigning appropriate contact models between particles to characterize the macroscopic mechanical response of granite.

Due to various modeling approaches, the computational principle between the bars and sample differs. The bars transmit velocity and displacement through grid cell nodes, while the sample transmits force and displacement through contacts between particles. To achieve interaction between the continuous and discrete domains, the wall zone command is used to generate coupled walls at the interface between the continuous and discrete domains. The coupling walls are split into a series of triangular faces connected by points and edges, which are then coordinated with the bar grid cell faces, achieving synchronous movement of the triangular face vertices and the FLAC model solid cell grid nodes. In each calculation cycle, the deformation of the continuous domain nodes drives the movement of the coupling walls, and the boundary node velocities are transmitted to the sample particles through the coupling walls. The sample particles follow the force–displacement law to calculate new contact forces and moments, and the centroid interpolation method is used to calculate equivalent forces and distribute them to each vertex of the coupling walls, realizing the interaction of force, velocity, and displacement between the continuous and discrete domains and enhancing the accuracy and efficiency of the simulation [28].

### 2.2. Jointed Granite Model

Granite, as a typical brittle material, has a microstructure composed of mineral crystals connected through strong bonding, requiring the selection of appropriate contact models based on meso-characteristics. Figure 2b indicates that the flat-jointed material, which can simulate the meso-structure of angular and interlocked particles, is frequently used for hard rock contact models with good results [29,30]. In Figure 2c, the flat-jointed model provides the macroscopic mechanical behavior of finite-sized linear elastic interfaces, which can exhibit bonded or frictional characteristics and can withstand partial damage. In 3D, the FJ model is a face contact, discretized into many elements along the radial ($N_{r}$) and circumferential ($N_{\alpha }$) directions. Each element can independently simulate bonding or sliding behavior, and each bonded element generates a microcrack when it fails, so a bond can produce up to $N_{r}\times N_{\alpha }$ microcracks, achieving local damage behavior of a single contact.

As shown in Figure 2d,e, bonded elements behave linearly elastically within the strength limit and become unbonded after exceeding the limit; unbonded elements exhibit linear elasticity and frictional characteristics, with sliding limited by applying the Coulomb limit to the shear force. Each element bears force and moment, following the force–displacement relationship, and the overall response displays the evolution from fully bonded to fully unbonded with frictional characteristics. When the tensile stress $\sigma^{\left(e\right)}$ or shear stress $\tau^{\left(e\right)}$ of a bonded element exceeds the specified tensile strength $\sigma_{c}^{\left(e\right)}$ or shear strength $\tau_{c}^{\left(e\right)}$ ($\tau_{c}^{\left(e\right)}=c-\sigma^{\left(e\right)}\tan\varphi$), it loses its bonding properties and turns into an unbonded state, where c and φ represent the cohesion and internal friction angle, respectively. Unbonded elements undergo frictional sliding when the shear stress $\tau^{\left(e\right)}$ exceeds the shear strength $\tau_{c}^{\left(e\right)}$ ($\tau_{c}^{\left(e\right)}=c_{r}-\mu \sigma^{\left(e\right)}$), where $c_{r}$ and μ represent the residual cohesion and friction coefficient, respectively; after which, the shear stress remains at this strength level. Materials using this model can undergo progressive damage at the micro level, enhancing the bending resistance with damage accumulation, and exhibit a high strength ratio under compression and tension.

**Figure 3:**
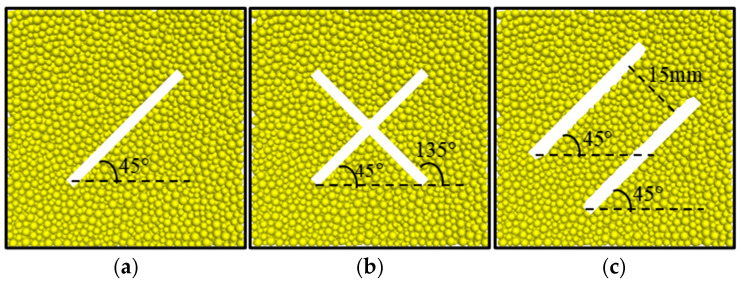
Joint setting scheme: (**a**) single joint; (**b**) cross joint; (**c**) parallel joint.

The intact granite rock sample has dimensions of 50 mm × 50 mm × 50 mm, composed of spherical particles with radii of approximately 0.6 mm to 1.0 mm. Figure 3 demonstrates the geometric configurations of three types of prefabricated jointed granite samples: (a) a single joint sample: a single penetrating joint at 45° to the impact direction; (b) a cross joint sample: primary and secondary joints perpendicular to each other at 45° and 135° to the impact direction, respectively; (c) a parallel joint sample: two joints both at 45° to the impact direction, with a spacing of 15 mm. All joints are generated using the particle deletion method, with a joint width of 0.6 mm, formed by removing particles in the specified area to create geometric weak planes. All joints are located in the central area of the sample, penetrating the entire sample in the vertical plane direction.

### 2.3. Multiaxial Hopkinson Pressure Bar

**Figure 4:**
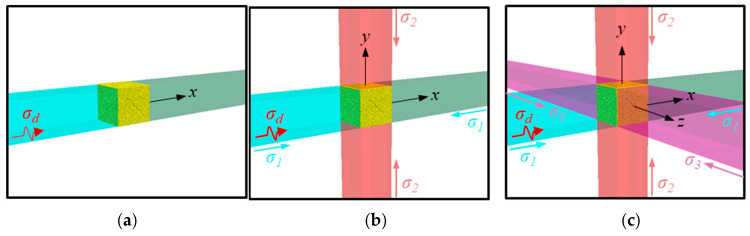
Setup of the (**a**) uniaxial, (**b**) biaxial, and (**c**) triaxial SHPB tests.

Figure 4 shows the modeling approach for the multiaxial preload loading system of the Hopkinson pressure bar experiment. For dynamic uniaxial compression (UC, $\left(\sigma_{1},\sigma_{2},\sigma_{3}\right)=\left(0,0,0\right)$ MPa), the incident and transmitted bars are aligned axially, simulating uniaxial dynamic compression through one-dimensional stress wave transmission. For dynamic biaxial compression (BC, $\left(\sigma_{1},\sigma_{2},\sigma_{3}\right)=\left(30,10,0\right)$ MPa), an output bar system in the Y-direction is added to the X-direction bar system, with the two sets of bars spatially orthogonal to form a biaxial preload loading system. For dynamic triaxial compression (TC, $\left(\sigma_{1},\sigma_{2},\sigma_{3}\right)=\left(30,20,10\right)$ MPa), an output bar system in the Z-direction is further added, with the three sets of bars mutually orthogonal to independently apply the preload in three directions. The incident bar is 2.5 m × 50 mm × 50 mm, and the output bars are consistent with the transmitted bar at 2.0 m × 50 mm × 50 mm, ensuring synchronism and coordination of multiaxial stress wave transmission through an equal cross-section design. In the numerical model, the steel bars use an isotropic elastic model with parameters strictly following the experimental calibration values: elastic modulus 200 GPa, Poisson’s ratio 0.27, and density 7850 kg/m^3^, matching the measured data of 60Si2Mn alloy steel used in experiments.

The biaxial and triaxial preload systems use normal face force boundary conditions to form the initial stress field. By applying the axial preload at the far ends of the orthogonal steel bars, the preload is transmitted to the central sample through the coupling walls to achieve stress equilibrium. During the dynamic loading phase, based on the time–history curve of the incident stress wave obtained by experimental measurement, the waveform data are mapped to the dynamic stress boundary condition at the end of the X-direction incident bar using the Table function built into FLAC.

### 2.4. Selection of Numerical Model Parameters

In the DEM framework, the FJ model drives the macroscopic mechanical response of the material through the bonding mechanical behavior between meso-scale particles. There is no explicit analytical relationship between the contact model and the macroscopic mechanical response, requiring a “trial and error” approach [31,32,33] to achieve cross-scale parameter calibration. Parameter calibration for the FJ model can significantly improve efficiency based on mature parameter sensitivity laws [26,27,34].

(1) Determination of the particle parameters: The minimum particle radius $R_{\min}$ depends on the balance between computational accuracy and efficiency, ensuring a sufficient number of particles across the specimen width. The maximum-to-minimum particle radius ratio $R_{\max}/R_{\min}$ is set to 1.66 to guarantee the stability of the particle system and meet the coordination number requirements. The density ρ settings conform to the actual physical properties of granite. (2) Determination of the deformation parameters: The strength parameters are fixed at relatively large values while progressively adjusting the effective modulus $E^{*}$ and normal-to-shear stiffness ratio $k^{*}$. The effective modulus $E^{*}$ primarily contributes to the macroscopic dynamic elastic modulus, while the normal-to-shear stiffness ratio $k^{*}$ influences the Poisson’s ratio of the specimen, which is reflected through the deformation in the Y-direction. (3) Determination of the strength parameters: The cohesion c and internal friction angle φ satisfy the Coulomb criterion. The tensile strength $\sigma_{c}$ and shear strength $\tau_{c}$ of the bonds govern the peak strength and failure mode of the specimen. (4) Determination of the meso-structural parameters: The bonds are installed at the ball–ball contacts with a gap g less than or equal to 0.3 times the minimum particle radius $R_{\min}$. The radial and circumferential element numbers ($N_{r}$ and $N_{\alpha }$) are set to 1 and 3, respectively. Table 1 provides a set of suitable parameters for the subsequent numerical model verification and analysis.

**Figure 5:**
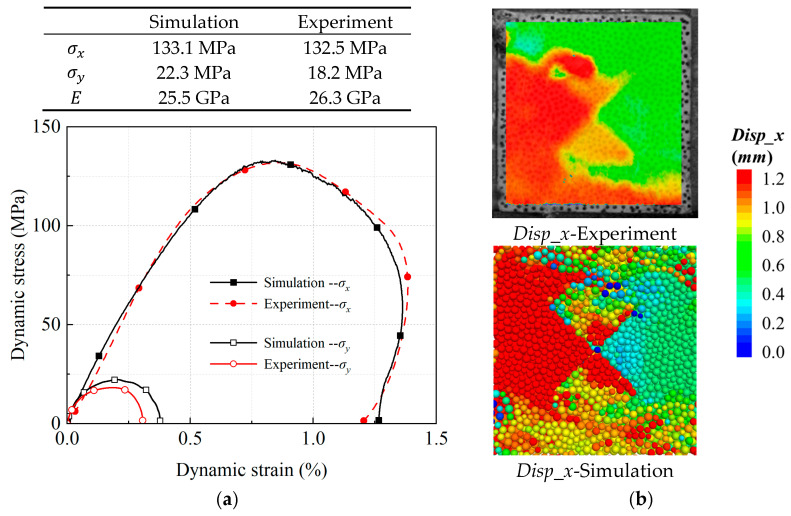
Comparison of experimental and simulated (**a**) dynamic stress–strain curves; (**b**) displacement field distributions.

Figure 5a presents the dynamic stress–strain curves of a cross joint specimen under an initial static load of $\left(\sigma_{1},\sigma_{2}\right)=\left(30,10\right)\;\mathrm{MPa}$ in a biaxial Hopkinson bar experimental system, compared with the actual experimental results. It can be observed that the dynamic stress–strain curves obtained from numerical simulations and physical experiments exhibit highly consistent evolutionary characteristics. In the impact direction (X-direction), the pre-peak curves show a quasi-linear increase. The simulated and experimentally measured dynamic elastic moduli are 25.5 Gpa and 26.3 Gpa, respectively. At a strain of 0.85%, the dynamic compressive strengths reach 133.1 Mpa (simulation) and 132.5 Mpa (experiment), both demonstrating smooth transition characteristics at the peak states. The post-peak curves decline gradually, with slight rebound phenomena observed when the stress decreases to 0.5 times the peak stress. In the confinement direction (Y-direction), the peak stresses and deformations, originating from the Poisson effect of the specimen, are significantly smaller than those in the impact direction. Both simulated and experimental dynamic stress–strain curves follow the evolutionary pattern of elastic deformation, peak failure, and residual rebound. Figure 5b displays the displacement field in the impact direction at 0.5 times the peak stress during the post-peak stage, measured through laboratory digital image correlation (DIC) techniques, alongside the particle displacement field obtained from numerical simulations. Both approaches demonstrate high consistency in the spatial distribution characteristics of displacement fields. The maximum displacement on the specimen surface reaches 1.2 mm, with the displacement field of the cross joint specimen in the impact direction exhibiting significant gradient differentiation and spatial heterogeneity. The consistent patterns revealed through curve comparisons and displacement field analyses indicate that the calibrated parameters in the constructed model effectively reflect the dynamic response processes observed in the actual experiments.

## 3. Model Verification and Result Analysis

### 3.1. Model Verification

**Figure 6:**
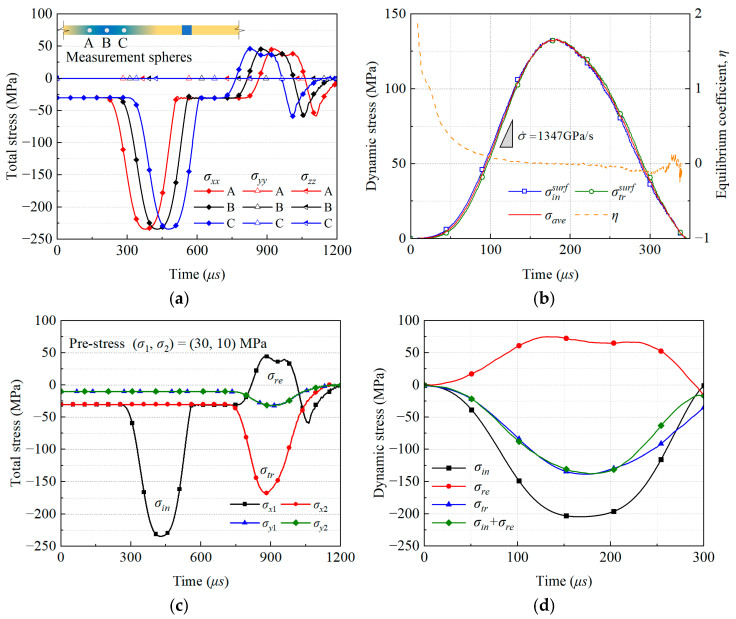
(**a**) Verification of the 1D stress wave assumption, (**b**) verification of the dynamic stress equilibrium, (**c**) stress–time histories in 4 bars, and (**d**) validation of three-wave method.

In the numerical simulation of SHPB, to verify the applicability of the one-dimensional stress wave propagation assumption, a biaxial loading system was employed to conduct static–dynamic composite loading tests on cross joint samples with an initial static load $\left(\sigma_{1},\sigma_{2}\right)=\left(30,10\right)\;\mathrm{MPa}$. Three monitoring points: A, B, and C were arranged along the central axis of the incident bar in the X-direction, located at 1.50 m, 1.25 m, and 1.00 m from the sample, respectively, as shown in Figure 6a, to collect the axial stress component ($\sigma_{\mathrm{xx}}$) and lateral stress components ($\sigma_{\mathrm{yy}}$ and $\sigma_{zz}$) in real time. The results show that the axial stress wave waveform remains highly consistent during propagation, with the axial stress values $\sigma_{\mathrm{xx}}$ measured at the three points increasing from the initial 30 MPa preload to the incident wave amplitude of 204.4 MPa over time, with synchronized phases and no significant attenuation, while the lateral stress components are negligible. This result confirms that the stress wave propagation in the numerical model strictly follows the one-dimensional propagation assumption.

To further verify the validity of the model, it is necessary to perform the dynamic stress equilibrium of the model through the “direct method” and the “three-wave method” to ensure that the sample is in a uniform stress field during dynamic loading. As shown in Figure 6b, by monitoring the stress $\sigma_{in}^{surf}$ at the sample–incident bar interface, the stress $\sigma_{tr}^{surf}$ at the sample–transmitted bar interface, and the average stress $\sigma_{ave}$ inside the sample in real time, the dynamic stress equilibrium coefficient $\eta =2\left(\sigma_{in}^{surf}-\sigma_{tr}^{surf}\right)/\left(\sigma_{in}^{surf}+\sigma_{tr}^{surf}\right)$ is defined to quantify the degree of dynamic stress equilibrium, where the magnitude of η reflects the relative difference between the dynamic stresses at both ends. In the initial stage, as the incident wave approaches the sample, the transmitted end has not yet responded, and η is 2; as the stress wave penetrates the sample, $\left|\eta \right|$ quickly decreases to less than 0.1, marking the entry of the model into the dynamic stress equilibrium stage; as the internal cracks in the sample expand and interfere with stress wave propagation, the difference between $\sigma_{in}^{surf}$ and $\sigma_{in}^{surf}$ increases, η oscillates and fails, and the dynamic loading process ends.

The stress wave histories in the four steel bars after impact are shown in Figure 6c, measured by monitoring points at the midpoints of the steel bars. Due to differences in initial pre-compression, the stresses $\sigma_{x1}$ and $\sigma_{x2}$ on the X-direction incident and transmitted bars start from 30 MPa, while $\sigma_{y1}$ and $\sigma_{y2}$ on the Y-direction-confined bars begin at 10 MPa. Subsequently, the stress waves propagate in the X-direction and undergo reflection and transmission through the specimen. Additionally, stress wave variations in the Y-direction are also monitored due to the Poisson effect of the specimen. Dynamic stresses were extracted from these raw stress signals by subtracting the static pre-stresses for further analysis. The three-wave method verifies stress equilibrium by analyzing the stress (strain) time–history curves of the incident wave, reflected wave, and transmitted wave. Figure 6d shows the result of stress equilibrium using the three-wave method, where it can be identified that the superposition of the incident wave and reflected wave extracted from the incident bar end ($\sigma_{in}+\sigma_{re}$) basically coincides with the transmitted wave extracted from the transmitted bar end ($\sigma_{tr}$). Through the “direct method” and the “three-wave method”, it is verified that the sample in the constructed numerical model is in a uniform stress state during dynamic loading, providing a reliable theoretical basis for the subsequent study of the dynamic mechanical behavior of jointed rock masses. Under the condition of stress equilibrium, the dynamic stress $\sigma_{i}$, dynamic strain $\varepsilon_{i}$, and strain rate ${\dot{\varepsilon }}_{i}$ in each direction of the sample can be obtained.

(1) $${\dot{\varepsilon }}_{x}\left(t\right)=\frac{C_{0}}{L}\left[\varepsilon_{in}\left(t\right)-\varepsilon_{re}\left(t\right)-\varepsilon_{tr}\left(t\right)\right]$$

(2) $$\varepsilon_{x}\left(t\right)=\frac{C_{0}}{L}\int_{0}^{t}\left[\varepsilon_{in}\left(t\right)-\varepsilon_{re}\left(t\right)-\varepsilon_{tr}\left(t\right)\right]dt$$

(3) $$\sigma_{x}\left(t\right)=\frac{E_{0}A_{0}}{2A_{s}}\left[\varepsilon_{in}\left(t\right)+\varepsilon_{re}\left(t\right)+\varepsilon_{tr}\left(t\right)\right]$$

(4) $$\varepsilon_{y}\left(t\right)=\frac{C_{0}}{L}\int_{0}^{t}\left[\varepsilon_{y1}\left(t\right)+\varepsilon_{y2}\left(t\right)\right]dt$$

(5) $$\sigma_{y}\left(t\right)=\frac{E_{0}A_{0}}{2A_{s}}\left[\varepsilon_{y1}\left(t\right)+\varepsilon_{y2}\left(t\right)\right]$$

(6) $$\varepsilon_{z}\left(t\right)=\frac{C_{0}}{L}\int_{0}^{t}\left[\varepsilon_{z1}\left(t\right)+\varepsilon_{z2}\left(t\right)\right]dt$$

(7) $$\sigma_{z}\left(t\right)=\frac{E_{0}A_{0}}{2A_{s}}\left[\varepsilon_{z1}\left(t\right)+\varepsilon_{z2}\left(t\right)\right]$$

where: $C_{0}$, $E_{0}$, and $A_{0}$ represent the longitudinal wave velocity, elastic modulus, and cross-sectional area of the bar, respectively; $A_{s}$ and L denote the initial cross-sectional area and length of the specimen; $\varepsilon_{in}\left(t\right)$, $\varepsilon_{re}\left(t\right)$, and $\varepsilon_{tr}\left(t\right)$ stand for the incident, reflected, and transmitted strain signals in the X-direction at time t; $\varepsilon_{y}\left(t\right)$ and $\varepsilon_{z}\left(t\right)$ correspond to the strain signals from the two output bars in the Y- and Z-directions at time t.

### 3.2. Result Comparison

To explore the coupled mechanism of joint spatial configuration and the initial stress state on the dynamic mechanical behavior of rock masses, this section systematically analyzes the dynamic stress–strain response characteristics of samples with different joint distributions (single joint, cross joint, and parallel joint) under uniaxial (UC), biaxial (BC), and triaxial (TC) initial stress paths through numerical simulation, determining the influence weights of the joint spatial configuration and initial stress state on the dynamic stress–strain curve.

**Figure 7:**
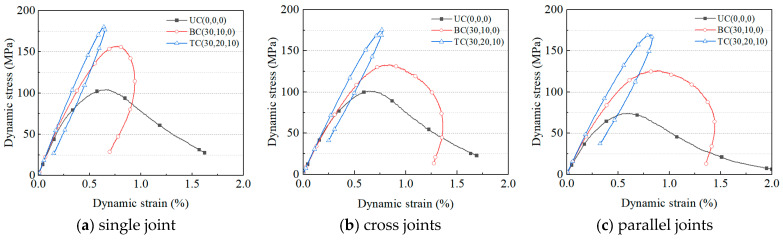

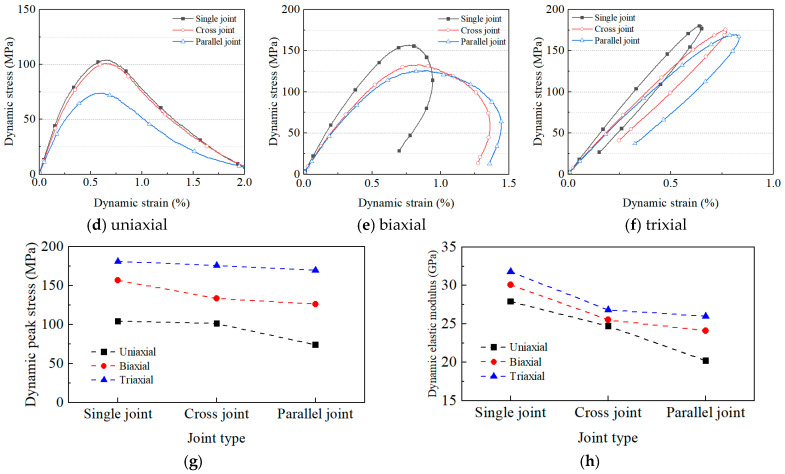
Dynamic stress–strain curves for (**a**) single joint, (**b**) cross joint, and (**c**) parallel joint specimens under different stress states. Dynamic stress–strain curves of three joint types under the (**d**) uniaxial, (**e**) biaxial, and (**f**) triaxial stress states. (**g**) Peak strength and (**h**) dynamic elastic modulus across joint configurations.

Figure 7a–c, based on the single joint configuration, compare the regulatory mechanisms of multiaxial stress states (UC/BC/TC) on the dynamic stress–strain response. The change in stress state significantly affects the mechanical properties of jointed samples subjected to impact. As the stress state transitions from uniaxial to triaxial, the curve morphology transitions from a gentle opening to a sharp hysteresis, with both peak stress and elastic modulus increasing, indicating that the initial constraint force on the model significantly enhances its resistance to deformation and failure. Compared with uniaxial stress conditions, the peak stress of single joint samples under biaxial and triaxial preload increases by 50.7% and 73.8%, respectively, and the dynamic elastic modulus increases by 7.9% and 14.0%, respectively; for cross joint samples, the peak stress increases by 31.8% and 73.9%, respectively, and the dynamic elastic modulus increases by 3.2% and 8.5%, respectively; for parallel joint samples, the peak stress increases by 70.8% and 129.4%, respectively, and the dynamic elastic modulus increases by 19.3% and 28.7%, respectively. Parallel joints exhibit the highest sensitivity to confining pressure, showing the largest relative increments in strength and stiffness under triaxial conditions.

Figure 7d–f focus on the fixed initial stress paths, comparing the differential effects of joint spatial distributions on the dynamic stress–strain response. Under each stress state, the mechanical performance of the three joint samples also varies. Under uniaxial stress, all three samples completely fail, with the curve shape showing a gentle opening. The peak stresses of single joint and cross joint samples are similar (104.1 MPa vs. 101.0 MPa) and significantly higher than that of the parallel joint sample (73.9 MPa). Under uniaxial stress conditions, the secondary joint in the cross joint sample has an insignificant effect on strength, while the secondary joint in the parallel joint significantly reduces the impact resistance of the sample. Under biaxial stress, the confining pressure effect becomes apparent, and the stress–strain curve evolves into a hysteresis type. The single joint sample has the smallest curve opening, showing obvious unloading and elastic recovery after the peak, while the cross and parallel joint curves have more divergent opening shapes. In terms of strength characteristics, the single joint sample maintains the highest peak stress (156.9 MPa), with the smallest peak strain (0.77%); the peak stresses (133.1 MPa vs. 126.2 MPa) and peak strains (0.85% vs. 0.89%) of the cross and parallel joint samples show convergence. Under triaxial confining pressure conditions, the peak strengths (180.9 MPa → 175.6 MPa → 169.5 MPa) and dynamic elastic moduli (31.8 GPa → 26.8 GPa → 26.0 GPa) of the single joint, cross joint, and parallel joint samples decrease sequentially, and the hysteresis opening of the stress–strain curve further shrinks compared to the biaxial state, forming a sharp hysteresis loop with a steep rising section. Figure 7g,h illustrate trends in the peak strength and elastic modulus under varying conditions, providing an intuitive basis for analyzing the synergistic effects of joint distribution and stress state on the mechanical properties. The mechanical response of jointed samples under confinement arises from the interplay between confining pressure constraints and joint spatial configuration. These factors jointly govern the dynamic behavior of rock masses by regulating crack initiation and propagation mechanisms.

**Figure 8:**
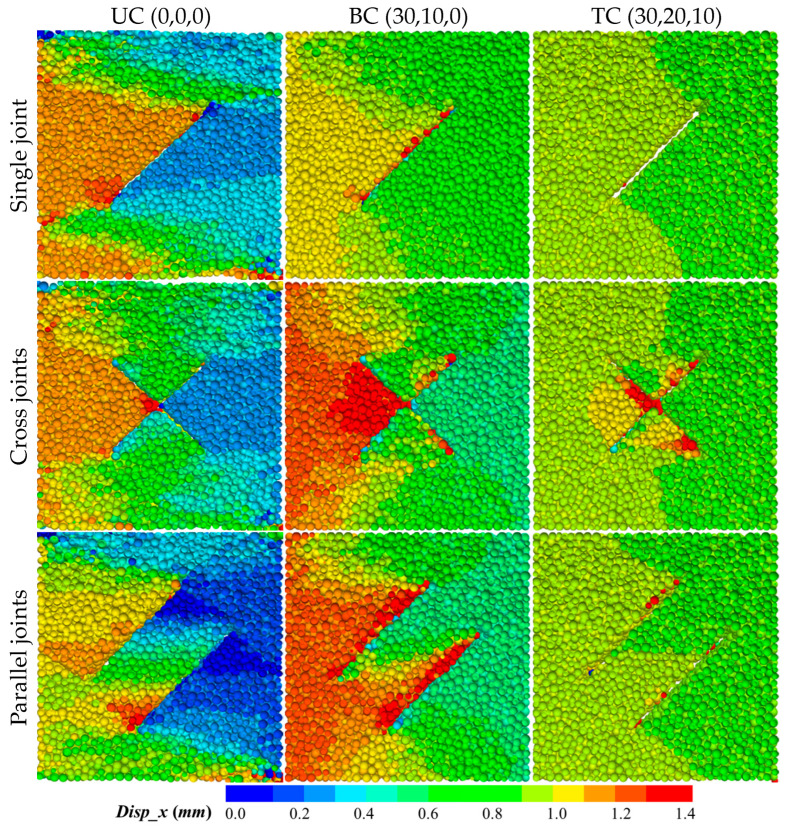
Distribution of the displacement field in the impact direction.

As the impact stress wave passes through the sample, particles are displaced, and the overall failure mode of the sample becomes apparent. Figure 8 shows the displacement field distribution along the impact direction XOY section of the sample when the stress drops to 0.2 times the peak strength for different joint types (single joint, cross joint, and parallel joint) and initial preload conditions (UC, BC, and TC), intuitively presenting the displacement magnitude through color transitions (blue to red, range 0.0 to 1.4 mm). The single joint sample exhibits the most significant displacement localization under uniaxial loading, with a clearly evident transition from blue (low displacement) to red (high displacement) in the displacement field. The displacement field is bounded by the existing joint plane on the left and right, and the upper and lower parts of the sample are also divided into different color transition zones. The boundaries of these various color transition zones in the displacement field are the channels for crack initiation, propagation, and extension. With increase in the lateral pressure (biaxial and triaxial loading), the red high-displacement area shrinks, and under triaxial loading, the displacement distribution is almost entirely green, with the displacement field gradually homogenizing and the localization trend significantly suppressed. Compared with the single joint sample, the displacement fields of the cross joint and parallel joint samples along the impact direction also exhibit pronounced gradient and spatial differentiation characteristics. The displacement field of the cross joint shows a unique bidirectional symmetric gradient distribution under the impact load, with the high-displacement area on the left side roughly equal in spatial extent to the low-displacement area on the right side, showing distinct mirror symmetry along the vertical direction. Although the displacement field distribution of the parallel joint system is similar in morphology to that of the single joint, a displacement transition zone is formed at the rock bridge due to the multilevel blocking effect of parallel joints. It is worth noting that the displacement values at the incident side and transmitted side of these two joint samples differ significantly under uniaxial preload conditions (e.g., cross joint incident side 1.2 mm vs. transmitted side 0.3 mm), presenting large non-uniformity. However, although the overall displacement values are higher than those under the uniaxial preload, the displacement difference between the incident side and transmitted side decreases (e.g., cross joint incident side 1.4 mm vs. transmitted side 0.6 mm) under the biaxial preload conditions. This difference reveals that biaxial constraints inhibit the accumulation of inelastic deformation through frictional locking effects, suppressing Poisson’s expansion of the material in non-impact directions, manifested as directional accumulation of displacement along the impact direction, and the sample maintains good integrity during impact, which explains the hysteresis change of the dynamic stress–strain curve from uniaxial to biaxial. The displacement fields of the cross joint and parallel joint samples under triaxial preload conditions show similar patterns to those under uniaxial conditions, with the displacement distribution gradually becoming uniform, localization trends significantly suppressed, and displacement values near the joints reaching the maximum. Overall, the displacement distribution of the sample gradually becomes holistic from the uniaxial to triaxial stress states, reflecting the significant enhancement of the sample’s impact resistance by the initial confining pressure; the directional expansion of a single weak plane is suppressed, maintaining high structural integrity, while the joint systems of the cross and parallel joint samples exhibit cooperative expansion effects, achieving greater inelastic deformation through crack propagation and penetration between joints.

## 4. Discussion

The impact load has a significant influence on the fracture behavior of jointed rocks, especially under high strain rates, where jointed rocks undergo complex progressive fracture processes. Using particle discrete element analysis, the mechanical responses of three different joint types of granite under biaxial stress states and cross joint granite under three initial stress states under impact loads are analyzed. This study compares the meso-fracture evolution laws of each condition through force chain distribution, contact force fabric, sample deformation, progressive crack propagation, and energy evolution.

### 4.1. Evolution of Contact Force Distribution

Force chains are networks formed by contact forces between particles, and in PFC software, they are the main function for internal stress transmission in materials. Force chains typically manifest as a series of interconnected particles with contact forces significantly higher than those between the surrounding particles. When external loads are applied, force chains bear the function of dispersing and transmitting external forces, thereby maintaining the overall stability of the material.

**Figure 9:**
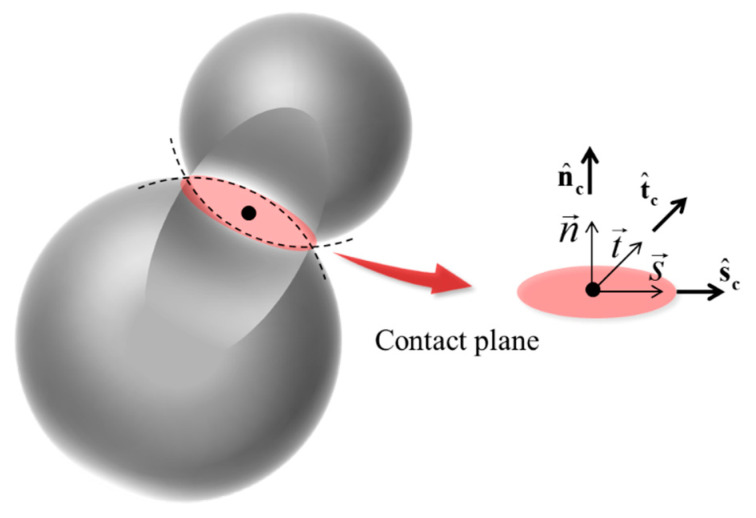
Contact force plane between particles.

As shown in Figure 9, contact between two particles in PFC forms a contact plane with a normal direction $\vec{n}$. A Cartesian coordinate system is established within the contact plane, ultimately forming a local coordinate system with $\vec{s}$, $\vec{t}$, and $\vec{n}$ as the basis. In this coordinate system, the contact force between particles can be decomposed into ${\hat{s}}_{c}$, ${\hat{t}}_{c}$, and ${\hat{n}}_{c}$. ${\hat{n}}_{c}$ represent the normal contact force. In the contact plane, the resultant of ${\hat{s}}_{c}$ and ${\hat{t}}_{c}$ denote the tangential contact force. The normal force is the skeleton of the force chain network, determining the compression state of the particles and the tightness of the contact. The formation, strength, and stability of the force chains are primarily governed by the normal contact force, while the tangential force mainly affects the sliding behavior and frictional characteristics of the particles.

**Figure 10:**
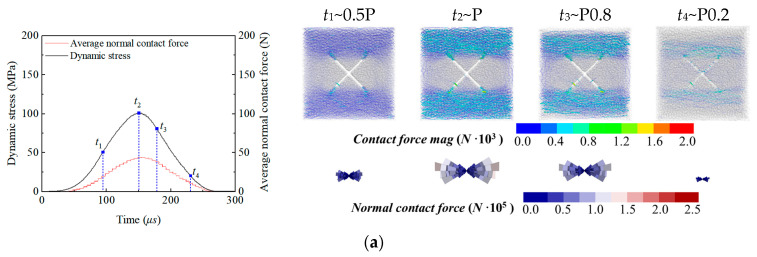

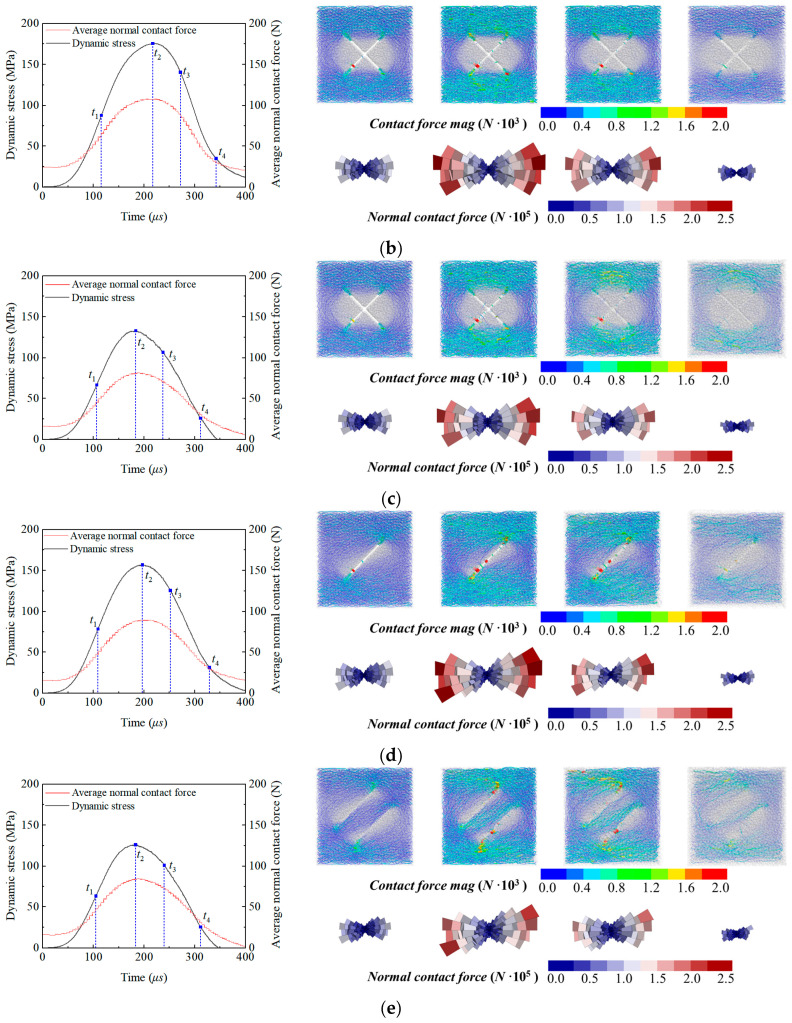
Average normal contact force evolution curve with the dynamic stress time history (left) and spatial distribution of force chains and normal contact fabric (right). (**a**) Uniaxial preloaded cross joint specimens, (**b**) triaxial preloaded cross joint specimens, (**c**) biaxial preloaded cross joint specimens, (**d**) biaxial preloaded single joint specimen, and (**e**) biaxial preloaded parallel joint specimens.

By traversing the normal contact forces of all contacts, the evolution trajectory of the average normal contact force with the stress time history during impact loading is plotted, as shown on the left side of Figure 10. It is evident that the average normal contact force of the sample shows strong consistency with the dynamic stress trend, indicating that the normal contact force directly reflects the stress state and is a key factor in the bearing capacity under impact loading. When the stress peaks, the average normal contact force achieves its maximum value (e.g., 108.2 N under the triaxial preload). The initial average normal contact force has a non-zero value under the biaxial/triaxial preload, reflecting the influence of the initial preload on particle contact. The right side of Figure 10 shows the evolution of force chains and normal contact force fabric during each impact loading process, and four key time points are selected for comparative analysis: 0.5 times the peak strength before the dynamic stress peak (*t*_1_), at the peak stress (*t*_2_), 0.8 times the peak strength after the dynamic stress peak (*t*_3_), and 0.2 times the peak strength after the dynamic stress peak (*t*_4_). The thickness of the force chains reflects the magnitude of the contact force, distinguished by a color scale. The normal contact force fabric characterizes the distribution of normal contact forces in various directions in space, revealing the main direction of the force chains (e.g., along the loading axis) and their strength differences, reflecting the stress transmission path of the material. The length and color depth of the bars in each direction interval represent the relative strength of the normal contact force in that direction under normalized scales. Comparing Figure 10a–c, it can be seen that, in the pre-peak stage (*t*_1_ moment, 0.5 times the peak strength), the main force chains concentrate at cross joint tips, forming stress concentration zones. Relatively thick main force chains are formed on the upper and lower sides of the sample, bearing the maximum principal stress distribution from the stress wave impact on the upper and lower sides, with the direction consistent with the impact direction. The left and right sides of the joint plane present force chain blank areas due to no constraints under uniaxial conditions, while there are secondary force chain networks under biaxial/triaxial conditions due to Y-direction constraints, with the main force chains being the thickest under triaxial conditions and second under biaxial conditions. At the peak stage (*t*_2_ moment), the main force chains on the upper and lower sides further thicken, and the radial length of the normal contact force fabric in the main direction (along the impact axis) reaches its maximum, with a deep red color, indicating that the stress transmission efficiency and bearing capacity reach their limits. In the post-peak damage stage (*t*_3_ moment, 0.8 times the peak strength), bond breakage leads to the degradation of some force chains, microcracks initiate along the joint tips, and the length and color depth of the main direction of the normal contact force fabric synchronously attenuate with damage accumulation. In the failure stage (*t*_4_ moment, 0.2 times the peak strength), the force chain network severely degrades, with residual force chains only bearing low-amplitude contact forces. The sample loses its bearing capacity due to the penetration of macroscopic cracks. Due to the significant constraining effect of the confining pressure, the sample retains some effective force chain networks under biaxial and triaxial stress states and maintains a certain residual strength and resistance to subsequent failure, while the force chain system under uniaxial conditions has significantly disintegrated due to the lack of confining pressure support, with only a very small amount of bearing force chains remaining. Comparing Figure 10c–e, since both single joint and parallel joint samples are arranged at 45°, the direction of force chains near the joints deflects due to geometric guidance, but thick main force chains are still formed on the upper and lower sides of the far end sample, with their direction consistent with the impact direction (horizontal direction). In parallel joint samples, secondary force chains appear at the rock bridge between joints, forming bridging bearing paths. The normal contact force fabric still shows a gradual increase trend before the peak and synchronously attenuating with damage accumulation after the peak. The main directions of the fabric for the single and parallel joint samples both deviate from the horizontal direction by about 30°, reflecting the anisotropy controlled by the joints.

### 4.2. Progressive Crack Propagation

**Figure 11:**
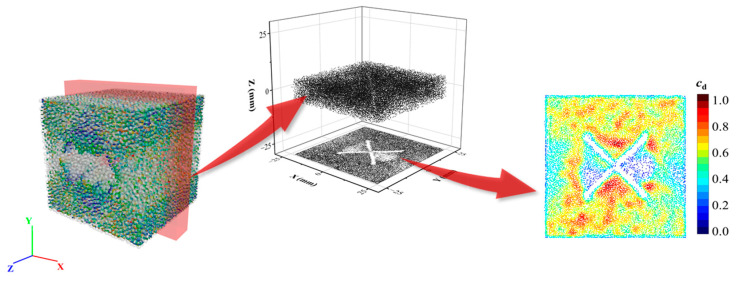
Crack drawing process.

Microcracks in discrete element modeling serve as a core concept for describing internal damage and progressive failure in materials. They are fundamentally characterized by the breakdown of interparticle bonds or contact failure, reflecting changes in the micromechanical behavior. The number, distribution, and evolution paths of the microcracks indicate the degree of internal damage within the material. Due to the unique “flange-like” bonding structure of the FJ model, where each bonded element (analogous to a bolt in a flange) generates a microcrack upon failure, the FJ model produces dense microcrack clusters during material failure. However, the three-dimensional (3D) spatial distribution of these microcracks is challenging to visualize intuitively. To optimize visualization, as illustrated in Figure 11, a 10-mm-thick central slice of the rock sample parallel to the XOY coordinate system is projected onto the XOY plane. A circular statistical window with a radius of 1.6 mm (equivalent to the average particle diameter in the model) is established, centered at each crack. The number of cracks per unit area is calculated as an absolute density index $c_{d}$, and all statistical data are normalized to obtain a continuous relative density field ranging from 0 (no cracks) to 1 (maximum density zone).

**Figure 12:**
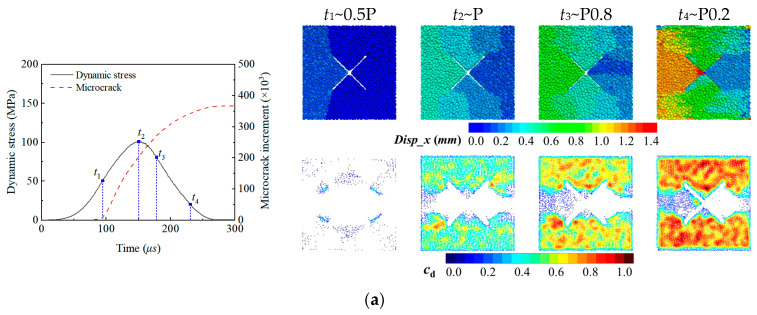

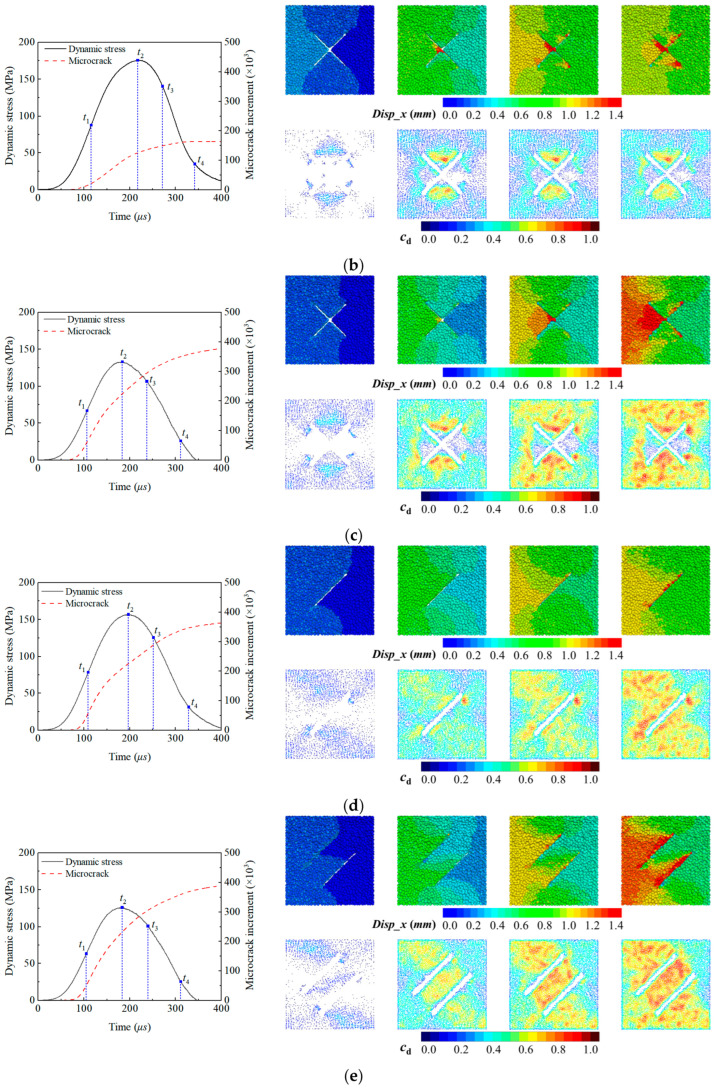
Evolution curve of microcrack numbers with dynamic stress history (left) and particle displacement and a microcrack relative density evolution map (right). (**a**) Uniaxial preloaded cross joint specimens, (**b**) triaxial preloaded cross joint specimens, (**c**) biaxial preloaded cross joint specimens, (**d**) biaxial preloaded single joint specimen, and (**e**) biaxial preloaded parallel joint specimens.

To investigate the influence of the initial stress state and joint spatial distribution on the dynamic failure process of rock, a multidimensional visualization approach is employed to reveal the underlying failure mechanisms. As demonstrated in Figure 12, the left side presents the evolution curve of the number of microcracks with dynamic stress history, while the right side integrates particle displacement cloud maps with microcrack relative density evolution maps. This visually illustrates the displacement field gradients, local deformation, crack density, and propagation paths within the rock at various moments, providing a comprehensive analysis of the synergistic control of the initial stress field and structural defects on dynamic failure modes.

As depicted in Figure 12a–c, cross-jointed specimens under different initial stress states are subjected to dynamic impact. Microcracks initiate at approximately 60 μs, and their number evolution exhibits a three-stage pattern. In the initial stage (<100 μs), a gentle curve slope appears due to low microcrack accumulation; in the rapid development stage (*t*_1_–*t*_3_), microcracks grow exponentially; and in the stable stage (>*t*_3_), the growth rate significantly slows, stabilizing by *t*_4_. The total number of microcracks in the biaxial specimens is comparable to that in the uniaxial specimens, while the triaxial specimens exhibit the fewest microcracks, less than half of the other two. Due to effect of the spatial distribution of X-shaped cross joints, the dynamic displacement field displays gradient evolution characteristics. The central region of the specimen forms displacement zones distributed at 90° intervals: the impact side (left) shows the largest displacement, the upper and lower regions are symmetrically distributed, and the transmission side (right) exhibits the smallest displacement, reflecting the retardation effect of the joint system on stress waves. Notably, the uniaxial and biaxial specimens exhibit significant displacement localization and gradient evolution. The biaxial specimens markedly suppress Poisson’s expansion in the non-impact direction, manifesting as directional displacement accumulation along the impact direction. In contrast, the uniaxial specimens experience premature overall failure due to multidirectional inelastic deformation, reducing the measured displacement in the impact direction. The confinement effect in the triaxial tests is more pronounced, resulting in a relatively uniform displacement field distribution and better preservation of the specimen integrity. Regarding joint density spatial distribution, cracks preferentially initiate at the wingtips of the four joint ends, forming arc-shaped rupture zones through reverse extension, followed by the crack network expanding fully toward the specimen boundaries. Crack density peaks induced by uniaxial conditions occur at the upper and lower ends of the specimen, while the left and right sides of the X-shaped joints (especially the transmission side) approach zero. Cracks distribute in biaxial specimens are more uniform around the joints due to Y-direction constraints, with reduced density at the upper and lower ends compared to uniaxial specimens. In triaxial specimens, crack localization is pronounced, concentrating in arc-shaped connection zones above and below the X-shaped joints, demonstrating the path-controlling effect of confining pressure on crack propagation.

As shown in Figure 12c,d, the microcrack evolution process remains largely consistent under biaxial initial stress states with different joint configurations subjected to dynamic impact. Parallel-jointed specimens exhibit slightly more microcracks, attributed to additional increments from secondary force chain fractures in rock bridges. In terms of displacement field distribution, single-jointed specimens form a clear left–right boundary along the preexisting joint plane, with displacement decreasing from the impact side to the transmission side. Parallel-jointed specimens, affected by multilevel joint retardation, develop gradual transition zones in the displacement field between adjacent joints. Single-jointed specimens present a more uniform displacement field and lower overall displacement compared to cross- and parallel-jointed specimens, due to suppressed directional extension along a single weak plane, maintaining a higher structural integrity. In contrast, the joint systems in the cross- and parallel-jointed specimens exhibit a synergistic extension effect. In all joint configurations, the initial damage begins with wing cracks at the joint tips. Due to effect of Y-direction constraint stress, microcrack-dense regions concentrate around joints and develop along layered interfaces within the displacement zones. Single-jointed specimens evolve into Z-shaped crack networks, and cross-jointed specimens induce multidirectional crack network expansion, significantly increasing the damage scope, while secondary force chain fractures between rock bridges in parallel-jointed specimens also contribute to localized microcrack-dense regions.

In summary, the dynamic failure process of rock is governed by the combined effects of the initial stress state and joint configuration. The initial stress level significantly regulates the scale and mode of failure, with rock damage transitioning from large-scale penetration to localized fragmentation as the lateral confinement increases from uniaxial to triaxial conditions. Confining pressure restricts the spread of damage zones by enhancing the frictional resistance at joint surfaces and suppressing crack propagation. The geometric constraints imposed by joint distribution patterns dominate the differentiation of failure paths, resulting in distinct crack propagation trajectories.

### 4.3. Energy Dissipation Law

Energy monitoring in the particle flow code (PFC) is a critical tool for analyzing the mechanical behavior of granular materials such as rock and soil. By tracking the evolution of various energy components, insights into material damage, energy transformation, and failure mechanisms can be obtained. Strain energy $E_{strain}$ stored in bonds and contacts between adjacent particles, frictional energy $E_{friction}$ generated by relative particle sliding, and kinetic energy $E_{kinetic}$ from particle motion can be directly monitored. The computational mechanisms for various energy components are defined as follows.

(8) $$E_{s}=\underset{\forall e}{\sum }E_{s}^{\left(e\right)}=\underset{\forall e}{\sum }\left(\frac{1}{2}\left(\frac{{\left(F_{n}^{\left(e\right)}\right)}^{2}}{k_{n}A^{\left(e\right)}}+\frac{{\left(F_{s}^{\left(e\right)}\right)}^{2}}{k_{s}A^{\left(e\right)}}+\frac{{\left(M_{b}^{\left(e\right)}\right)}^{2}}{k_{n}I^{\left(e\right)}}+\frac{{\left(M_{t}^{\left(e\right)}\right)}^{2}}{k_{s}J^{\left(e\right)}}\right)\right)$$

where $F_{n}^{\left(e\right)}$ and $F_{s}^{\left(e\right)}$ represent the normal and tangential forces acting on the element; $k_{n}$ and $k_{s}$ denote the normal and tangential stiffness of the bond; $M_{b}^{\left(e\right)}$ and $M_{t}^{\left(e\right)}$ are the bending moment and twisting moment applied to the element; $I^{\left(e\right)}$ and $J^{\left(e\right)}$ correspond to the moment of inertia and polar moment of inertia of the element; $A^{\left(e\right)}$ indicates the cross-sectional area of the element.

(9) $$E_{f}=\underset{\forall e}{\sum }E_{f}^{\left(e\right)}=\underset{\forall e}{\sum }\left(E_{f}^{\left(e\right)}+\tau_{c}^{\left(e\right)}A^{\left(e\right)}\|\Delta {\vec{\delta }}_{s}^{\left(e\right)}\|\right)$$

where $\tau_{c}^{\left(e\right)}$ represents the shear strength of the element; $\Delta {\vec{\delta }}_{s}^{\left(e\right)}$ denotes the relative shear displacement increment.

(10) $$E_{k}=\frac{1}{2}\underset{s}{\sum }(m_{i}v_{i}^{2})$$

where $m_{i}$ defines the generalized mass; $v_{i}$ corresponds to the generalized velocity.

**Figure 13:**
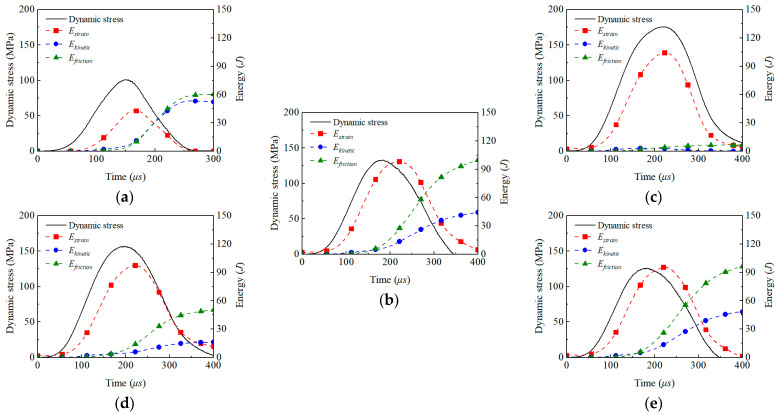
Dynamic stress and energy partition. (**a**) Uniaxial preloaded cross joint specimens, (**b**) biaxial preloaded cross joint specimens, (**c**) triaxial preloaded cross joint specimens, (**d**) biaxial preloaded single joint specimen, and (**e**) biaxial preloaded parallel joint specimens.

Figure 13a–c compares the energy evolution with the stress history for cross-jointed specimens under uniaxial, biaxial, and triaxial dynamic–static combined loading. During dynamic stress loading, the strain energy exhibits a pattern of initial accumulation followed by release; strain energy accumulates continuously through elastic deformation as the dynamic stress increases, then gradually releases post-peak stress due to specimen failure or an unloading rebound. Compared to the uniaxial state, biaxial and triaxial specimens influenced by initial preloading begin accumulating strain energy from a non-zero baseline, with the peak strain energy reaching 2.28 and 2.41 times that of the uniaxial case, respectively. Alongside strain energy evolution, kinetic and frictional energy increase synchronously with impact-induced deformation, significantly accelerating post-peak stress due to rapid crack propagation and stabilizing as loading ceases. A key difference between the biaxial and triaxial states lies in the energy dissipation mechanisms: biaxial specimens, with a free deformation surface, exhibit substantially higher kinetic and frictional energy than triaxial specimens. Figure 13b,d,e compare the energy evolution under biaxial dynamic–static combined loading for single-jointed, cross-jointed, and parallel-jointed specimens. Under identical initial preloading and stress wave conditions, the strain energy accumulation phase is highly consistent across all joint types, showing linear growth with dynamic stress, followed by synchronous release post-peak due to specimen rebound or rupture. However, energy dissipation mechanisms diverge significantly. Single-jointed specimens, with a higher peak dynamic stress and limited crack scale, exhibit notably lower increments in frictional and kinetic energy compared to cross- and parallel-jointed specimens. The latter two develop complex energy dissipation paths through multidirectional crack propagation or rock bridge fractures, promoting the sustained accumulation of frictional and kinetic energy.

## 5. Conclusions

Based on the coupled FDM and DEM, this study establishes a 3D numerical model of jointed rock specimens under a split Hopkinson pressure bar (SHPB) system from uniaxial to triaxial loading. The effects of joint configuration and stress state on the dynamic response of rock are investigated. The following conclusions can be drawn:

(1) Initial preloading and joint effects: Initial preloading significantly improves the rock impact resistance, but secondary joints weaken the load-bearing capacity through stress redistribution. Single-jointed specimens exhibit superior mechanical properties due to the structural integrity, while parallel-jointed specimens present the most pronounced strength reduction due to the stress concentration between joints. Triaxial conditions yield the highest relative increases in strength and stiffness, whereas cross-jointed specimens influence the load-bearing capacity by altering the deformation directions.

(2) Dynamic stress propagation: The stress propagation process exhibits three stages: Mesoscopic force chains align directionally during the stress accumulation phase, coarse primary force chains form around joint tips at peak intensification, and force chain degradation triggers crack propagation during damage dissipation. The primary force chain network is densest under triaxial stress. Single-jointed specimens show the longest extension of primary force chains in the dominant direction, while cross-jointed specimens display uniform force chain distribution, and parallel-jointed counterparts exhibit clustering between joints.

(3) Failure mode transition: Rock failure transitions from large-scale fracture penetration to localized fragmentation as the lateral confinement increases from uniaxial to triaxial. All joint types initiate damage with wing cracks at the joint tips. Single-jointed specimens develop Z-shaped crack networks, cross-jointed specimens induce multidirectional crack networks that significantly expand the damage scope, and parallel-jointed specimens enhance crack connectivity between joints to form rock bridge structures, highlighting the directional control of joint spatial distribution on failure paths.

(4) Energy characteristics: Multiaxial confinement enhances the strain energy storage capacity and reduces the kinetic energy dissipation. Single-jointed specimens exhibit optimal energy storage, cross-jointed specimens enhance multidirectional energy dissipation via stress wave scattering, and parallel-jointed specimens concentrate on energy dissipation along dominant joint paths, forming distinct energy transformation modes.

(5) This study elucidates the regulatory mechanisms governing the dynamic response of rock masses through the synergistic–competitive interactions between joint spatial configurations and multiaxial stress states. It provides theoretical support for dynamic stability assessment and instability early warning in deep underground engineering, the prediction of crack propagation along joint-dominated paths, and the optimization of support system spatial arrangements and dynamic control strategies. However, the current work does not account for multi-physics coupling effects under extreme conditions (e.g., thermo-hydro-mechanical (THM) coupling in geothermal extraction involving high temperatures and seepage pressures), where rock mass damage mechanisms may exhibit significant deviations. To address this limitation, future research will develop a FDM-DEM hybrid model incorporating thermo-mechanical seepage coupling, aiming to elucidate the dynamic damage evolution and energy competition mechanisms under multi-field coupling effects, thereby enhancing the predictive framework for rock mass dynamic responses in deep engineering applications.

(6) This study aims to elucidate the coupled interaction mechanisms between initial stress fields and joint structural characteristics in governing the dynamic mechanical behavior of rock specimens. A subsequent experimental data analysis will employ multivariate analysis of variance and regression modeling as the core statistical inference methodologies. The investigation will specifically focus on deciphering the interactive effects of stress levels and joint geometry parameters across three key mechanical response dimensions: dynamic strength evolution, energy dissipation patterns, and failure characteristics. A statistical significance evaluation framework will be established to quantitatively characterize the competitive weightings of controlling factors and their synergistic interaction patterns in dynamic load-bearing systems.

## Figures and Tables

**Table 1:** Parameter settings for the numerical models.

Parameter	Value	Symbol (Unit)
***Particles*** *		
Minimum particle radius	0.6	*R*_min_ (mm)
Particle radius ratio	1.66	*R*_max_/*R*_min_
Density	2700	*ρ* (kg/m^3^)
***Contacts (FJ Model)*** *		
Number of elements in radial direction	1	*N_r_*
Number of elements in circumferential direction	3	*N_α_*
Installation gap	0.18	*g* (mm)
Effective modulus	43.0	$E^{*}$ (GPa)
Normal-to-shear stiffness ratio	3.0	$k^{*}$
Tensile strength	26.0	*σ_c_* (MPa)
Cohesion	130.0	*c* (MPa)
Friction angle	45	*φ* (°)
Friction coefficient	0.45	*μ*
***Bar system (Elastic Model)*** *		
Young’s modulus	200.0	*E* (GPa)
Poisson	0.27	*ν*
Density	7850	*ρ_s_* (kg/m^3^)

* The parameters belong to the ***Particles***, ***Contacts***, and ***Bar system*** components of the model.

## Data Availability

The original contributions presented in this study are included in the article. Further inquiries can be directed to the corresponding author.
